# Separation of functionally divergent muscle precursor cell populations from porcine juvenile muscles by discontinuous Percoll density gradient centrifugation

**DOI:** 10.1186/s12860-018-0156-1

**Published:** 2018-03-09

**Authors:** Claudia Miersch, Katja Stange, Monika Röntgen

**Affiliations:** Leibniz Institute for Farm Animal Biology (FBN), Institute of Muscle Biology and Growth, Growth and Development Unit, Wilhelm-Stahl-Allee 2, 18196 Dummerstorf, Germany

**Keywords:** Pig, Satellite cells, Myogenic marker expression, Functional heterogeneity, Primary muscle cell culture, Differentiation

## Abstract

**Background:**

Satellite cells (SC) and their descendants, muscle precursor cells (MPC), play a key role in postnatal muscle development, regeneration, and plasticity. Several studies have provided evidence that SC and MPC represent a heterogeneous population differing in their biochemical and functional properties. The identification and characterization of functionally divergent SC subpopulations should help to reveal the precise involvement of SC/MPC in these myogenic processes. The aim of the present work was therefore to separate SC subpopulations by using Percoll gradients and to characterize their myogenic marker profiles and their functional properties (adhesion, proliferation, and differentiation).

**Results:**

SC/MPC from muscles of 4-day-old piglets were isolated by trypsin digestion and enriched by Percoll density gradient centrifugation. A mixed myogenic cell population was obtained from the 40/70% interface (termed: mixed P40/70) of a 25/40/70% Percoll gradient. Thereafter, by using a more stepped 25/40/50/70% Percoll gradient, mixed P40/70 was divided into subpopulation 40/50 (SP40/50) collected from the 40/50% interface and subpopulation 50/70 (SP50/70) collected from the 50/70% interface.

All three isolated populations proliferated and showed a myogenic phenotype characterized by the ability to express myogenic markers (Pax7, MyoD1, Desmin, and MyoG) and to differentiate into myotubes. However, compared with mixed P40/70, SP40/50 and SP50/70 exhibited distinct functional behavior. Growth kinetic curves over 90 h obtained by the xCELLigence system and proliferation assays revealed that SP40/50 and mixed P40/70 constituted a fast adhering and fast proliferating phenotype. In contrast, SP50/70 showed considerably slower adhesion and proliferation. The fast-proliferating SP40/50 showed the highest myogenic differentiation potential with higher fusion rates and the formation of more middle-sized and large myotubes.

**Conclusions:**

The described Percoll density gradient centrifugation represents a useful tool for subdividing pig SC/MPC populations with divergent myogenic functions. The physiological role of SC subpopulations during myogenesis and the interaction of these populations can now be analyzed to a greater extent, shedding light on postnatal growth variations in pigs and probably in other animals.

## Background

Skeletal muscles are composed of myofibers, mainly formed during prenatal development by embryonic and fetal myoblast populations [1, 2]. Only after the establishment of primary and secondary myofibers can so-called satellite cells (SC) be morphologically defined by their typical position between the basal lamina and the sarcolemma of the muscle fibers [3, 4]. During peri- and postnatal growth, 30–60% of the nuclei in porcine muscle are proliferating SC [5], and a high proportion of their progeny differentiates to fuse with adjacent myofibers thereby providing the main source of new myonuclei [6–8]. In this rapid growth phase, the number of SC constantly declines representing 2–5% of nuclei in adult muscle [9, 10]. Simultaneously, increasing numbers of SC exit the cell cycle to form the adult quiescent stem cell pool. Quiescent SC are able to re-enter the cell cycle in response to activating stimuli (e.g., injury or physical activity) to participate in muscle maintenance and regeneration [11, 12]. During the quiescent phase, SC express the transcription factors paired box 7 (Pax7) and myogenic factor 5 (Myf5) [13–15]. About 10% of SC never express Myf5 (Pax7^+^/Myf5^−^ cells) and are thought to represent the “true” adult satellite stem cell pool [13]. Activated and proliferating SC are characterized by the expression of MyoD1 [16–18]. Both Myf5 and MyoD are important in determining muscle cell fate, either by regulating a return to the quiescent state to renew the SC pool or by initiating cellular differentiation accompanied by Pax7 downregulation and the induction of the early differentiation marker Myogenin (MyoG) [15, 19]. Terminally differentiated cells express sarcomeric myosin heavy chain (MHC) proteins [1, 20]. These cells fuse with each other to form nascent myotubes and/or fuse to differentiated muscle fibers [9].

In most studies, SC, myogenic precursor cells (MPC), and myoblasts are obtained from skeletal muscle by the dissociation of whole muscle by using mechanical procedures combined with enzymatic digestion [2, 21]. However, these isolates/cultures contain myofibrillar debris and are a mixture of myogenic and non-myogenic cells, mostly erythrocytes and fibroblasts [21–23]. This cellular impurity can be reduced by the use of enrichment procedures such as fluorescence-activated cell sorting [24], differential centrifugation [25], the preplate technique [25–27], or Percoll density gradient centrifugation [28–30]. In the Percoll density gradient fractionation system, several layers of solutions with different Percoll concentrations and, thus, densities are used to separate myogenic cells from non-myogenic cells [23, 31]. After centrifugation, cell fractions having different densities appear at the interfaces between the Percoll layers and can be collected. However, density gradient centrifugation is rarely used to separate the enriched myogenic cells further into functionally divergent subpopulations, despite this technique having been originally established to separate mononuclear blood cell populations [32]. In a study of Che et al. [31], an association was postulated between cell density and cell maturity as measured via molecular marker expression, but no information was provided about the functional properties of the separated cell populations.

Accumulating evidence indicates that SC populations are heterogeneous not only because of differences in their myogenic marker expression, but also because of their developmental stage (fetal, postnatal, adult) [1, 33, 34] and their myogenic function [35, 36]. In the postnatal period, nearly 80% of SC/MPC are highly proliferative [5, 37, 38], whereas the remaining cells divide slowly [37, 39] and retain stem cell properties for long-term self-renewal [40].

To date, several methods have been described to isolate and cultivate porcine SC and MPC [23, 41–44], but the heterogeneity of these cells, particularly during the postnatal growth phase, has rarely been considered so far. One reason for this is the lack of an effective but simple method to separate distinct myogenic cell populations by any other way than analyzing them for myogenic marker expression. The investigation of the heterogeneity of mixed (mass) cultures of SC/MPC and myoblasts is however a prerequisite for the better understanding of the origins and targeted modulation of growth phenotypes and of certain pathological states. Recently, we have shown that the early postnatal development of functionally heterogeneous SC subpopulations is associated with the bioenergetic profile of these cells [45]. In the present study, we describe an adequate method for separating SC subpopulations by Percoll density gradient centrifugation enabling the investigation of the heterogeneity and interaction of SC/MPC by the examination the myogenic properties of the mixed myogenic population in comparison with the divided subpopulations.

## Methods

### Animals

The cells for the experiments were obtained from 4-day-old German Landrace piglets having a normal birth weight (1.20 ± 0.25 kg) and raised in the experimental pig unit of the Leibniz Institute for Farm Animal Biology (FBN). Animal husbandry and slaughter followed the guidelines set by the Animal Care Committee of the State Mecklenburg-Western Pomerania, Germany, based on the German Law of Animal Protection.

### Isolation and cultivation of myogenic cells

The right and left *Musculus semimembranosus* (SM) and the right and left *Musculus longissimus dorsi* (LD) were removed in one piece, trimmed of visible connective tissue, and weighed in phosphate-buffered saline (PBS) containing 25 mM glucose, 14 mM sucrose, 1000 U/ml penicillin (PAN Biotech), 1 mg/ml streptomycin (PAN Biotech), and 25 μg/ml amphotericin (PAN Biotech).

Dissected muscle tissue was washed and minced intensively with scissors before fractionated enzymatic digestion was performed for 2 × 30 min with 1× trypsin solution (0.25%, 4000 U/ml, Sigma Aldrich) in a water bath with stirring at 37 °C. After being washed and filtered through gauze and fine nylon mesh (20 μm), muscle-dissociated cells were subjected to Percoll (Sigma Aldrich) gradient density centrifugation (1800 x g for 1 h). The fractionated cells were re-suspended in growth medium (αMEM Eagle, 20% FBS, 100 U/ml penicillin/streptomycin, 2.5 μg/ml amphotericin, and 0.05 mg/ml gentamycin; all from PAN Biotech).

**Fig. 1:**
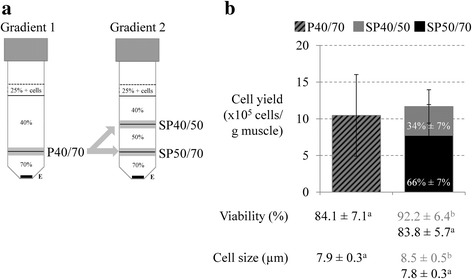
Overview of Percoll gradients employed and cell characteristics directly after isolation. **a** Trimmed muscle fragments from SM and LD muscle of 4-day-old piglets were digested with 0.25% trypsin and enriched at different Percoll layers by density gradient centrifugation. The collected cell populations are marked in gray, the erythrocyte fraction in black (E). A mixed myogenic population was isolated from the 40/70% interface (mixed P40/70) of a 25%, 40%, and 70% Percoll gradient and was separated into 40/50% and 50/70% subpopulations (SP40/50 and SP50/70) by the use of a second Percoll gradient with 25%, 40%, 50%, and 70% layers. **b** Cell yield, viability, and cell size of isolated cells from SM and LD muscle were determined, directly after isolation, with the Countess Automated Cell Counter. All data represent means ± SD, *n* ≥ 7 animals. The cell yield from SP40/50 and SP50/70 together did not differ from mixed P40/70, but SP40/50 showed a higher cell viability and cell size. A t-test was performed for the statistical analysis of differences in cell yield between mixed P40/70 and both subpopulations together. For the statistical analysis of cell viability, a Kruskal-Wallis One-Way ANOVA on Ranks was performed following a pairwise multiple comparison procedure (Dunn’s method). An ANOVA with the Holm-Sidak post-hoc test was performed for the statistical analysis of cell size. Letters mark significant differences within the populations (*p* ≤ 0.05)

Cell dissociation and digestion were performed as described by Mau et al. [23], but the employed Percoll density gradients were modified. Mau et al. [23] utilized a 25%, 40%, and 90% Percoll gradient to enrich SC/MPC at the 40/90% interface. The cell isolates obtained with this gradient were free from myofibril fragments, debris, and fibroblasts but were highly contaminated with erythrocytes. Therefore, as described by Bischoff and Heintz [46], the 90% Percoll layer was substituted by a 70% Percoll layer in our study. This procedure resulted in a clear separation of MPC from erythrocytes, which were now mainly found at the bottom of the tube. To isolate distinct SC/MPC subpopulations, two Percoll gradients composed of 25%, 40%, and 70% (gradient 1) or 25%, 40%, 50%, and 70% (gradient 2) Percoll layers were used. When gradient 1 was used, cells were taken from the 40/70% interface and termed mixed population 40/70 (mixed P40/70). When gradient 2 was used, mixed P40/70 was divided into 40/50 and 50/70 subpopulations (SP40/50 and SP50/70) that were obtained from the 40/50% and 50/70% interfaces, respectively (see Fig. 1a). Cells were seeded at approximately 0.5 × 10^6^ cells/cm^2^ in dishes coated with collagen type I (Greiner Bio-one) and cultured under a humidified atmosphere with 5% CO_2_ at 37 °C. At 24 h after seeding, the cells were washed with PBS containing 100 U/ml penicillin/streptomycin, 2.5 μg/ml amphotericin, and 0.05 mg/ml gentamycin. Bacterial and fungal contamination of cells was tested via inoculation of CASO Bouillon Tryptic Soy Broth (Merck) and Thioglycolate medium EP (Merck).

For passaging, cultured SC/MPC were detached by using HyClone HyQTase (Thermo Fisher Scientific), and the reaction was stopped by adding PBS containing 10% FBS. After centrifugation (10 min, 300 g, 10 °C), cells were re-suspended in growth medium, and the cell number was determined. From the first passage on, SC were cultured on Primaria® tissue culture dishes (VWR International) in growth medium.

### Determination of cell number, cell size, and viability

Cell number, cell size, and viability were quantified by using the Countess Automated Cell Counter (Thermo Fisher Scientific) following the manufacturer’s instructions. This system utilizes an image analysis algorithm combined with trypan blue staining to obtain accurate counts of cells, cell size, and viability.

### Analysis of myogenic marker expression with flow cytometry

Cultured SC were detached at day 4 and day 8 after isolation by using HyClone HyQTase, and the reaction was stopped by adding PBS containing 10% FBS. After centrifugation (10 min, 300 g, 10 °C), cells were re-suspended in 1 mM EDTA in PBS and fixed in 4% paraformaldehyde (PFA). Following fixation, cells were permeabilized with 0.1% (Desmin, MyoG) or 0.5% (Pax7, MyoD1) Triton X-100 in PBS, blocked with normal rabbit serum, and incubated overnight with the respective primary antibody: mouse anti-Desmin (clone D-33, DAKO, 1:80), mouse anti-Pax7 (Developmental Studies Hydridoma Bank, 1:50), mouse anti-MyoG (clone F5D, abcam, 1:50), or mouse anti-MyoD1 (clone 5.2F, abcam, 1:200). A portion of each sample was incubated with normal rabbit serum instead of primary antibody as a negative control. After two additional washing steps, samples were incubated with a rabbit anti-mouse Alexa488 antibody (Thermo Fisher Scientific, 1:1000) for 1 h at room temperature and washed again. In total, 5000 events per sample were analyzed by using an argon-equipped Gallios Flow Cytometer (Beckman Coulter) and evaluated with Kaluza software (Beckman Coulter).

### Proliferation rate

To determine cell proliferation rate, cells were seeded at similar densities (3-4 × 10^5^ cells) on Primaria® tissue culture dishes, and the cell number was determined after passaging by using the Countess Automated Cell Counter. Fold change was calculated as the difference between the final and the initial cell number.

### Impedance-based measurement of cell adhesion and proliferation

The xCELLigence (RTCA-SP, ACEA Biosciences Inc.) was used according to the manufacturer’s instructions for the continuous real-time monitoring of cell adhesion and proliferation by cell-electrode impedance [47] displayed as the dimensionless Cell Index (CI). By using an RTCA Analyzer, electrical impedance changes were measured across interdigitated microelectrodes integrated into the bottom of a specialized 96-well plate (E-Plate® 96) and sent to the RTCA control subunit. The latter used the RTCA software (version 2.0) for CI calculations from the frequency-dependent electrode resistances and real-time display of data. Specific details of the xCELLigence technology are described by Atienza et al. [48] and Ke et al. [49].

The background impedance of E-Plate® 96 was determined with growth medium only. Subsequently, 5000 or 40,000 cells per well were plated in a final volume of 200 μl growth medium. Based on initial growth curve experiments (data not shown), a high cell density of 40,000 cells per well was chosen to analyze cell adhesion (adhesion assay). In order to enable exponential growth, a low cell density of 5000 cells per well was used to examine the proliferation capacity of cells (proliferation assay). The cells were seeded in the E-Plate® 96, the plate was placed into a CO_2_-incubator, and the CI was monitored every 15 min over a period of 90 h. Cells were provided with fresh medium after 48 h. The RTCA software was used to calculate growth parameters: the slope during the cell adhesion phase (ΔCI/Δtime), CI decrease after medium change, the doubling time (DT) during the logarithmic growth phase, and the time to reach half of the maximal CI value (50% of CI_max_) during the experiment. In all experiments, each subpopulation was measured in quadruplicate, and experiments were repeated at least 5 times with cells from different animals.

### Differentiation assay

For myogenic differentiation, cells were seeded directly after isolation (mixed P40/70 and SP40/50: 1.5 × 10^5^ cells/well; SP50/70: 3 × 10^5^ cells/well) on Primaria® 24-well plates coated with Matrigel (growth factor reduced; 1:50, BD Biosciences) and cultivated in growth medium. At 80–90% confluency, cells were washed with PBS to remove excessive serum, and differentiation medium with 2% FCS was added. Medium was changed regularly. Finally, cells were washed with PBS and fixed in 4% PFA. A Nikon Diaphot 300 microscope and Olympus cell^F^ software were used to examine cells light microscopically.

### Immunofluorescence and quantification of differentiation

The fixed and differentiated cells were permeabilized with 0.1% (Desmin) or 0.3% (MHC) Triton X-100, blocked with normal rabbit serum, and incubated with the primary antibody, namely mouse anti-Desmin (clone D-33, DAKO, 1:80) or mouse anti-skeletal fast Myosin (MHC; clone MY-32, Sigma Aldrich, 1:400), overnight. After being washed with PBS, the samples were incubated with a rabbit anti-mouse Alexa488 antibody (Thermo Fisher Scientific, 1:1000) for 1 h at room temperature, and subsequently, cell nuclei were stained with DAPI (15 min, 1 μg/ml). For fluorescence microscopy, the Nikon Diaphot 300 microscope and Olympus cell^F^ software were used. Micrographs were merged by using Adobe Photoshop CC 2017; contrast and brightness were adjusted to the same degree in every sample group.

MHC+ myotubes containing ≥2 nuclei were encircled to determine the myotube area by using ImageJ 1.49v. For each experiment, 10–12 random sections per population were analyzed. Micrographs of the Desmin-stained cells were used to determine cell fusion rate. The number of nuclei in the fused multinucleated cells (≥ 2 nuclei) was divided by the total number of visible nuclei in 5 random sections for each experiment and subpopulation. Experiments were repeated independently three times with cells obtained from three animals.

### Statistical analysis

**Fig. 2:**
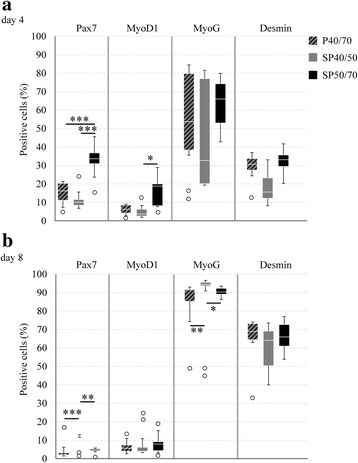
Myogenic marker expression of isolated SC/MPC populations. Flow cytometric analysis of immunofluorescence-stained SC/MPC from SM and LD muscle cultured for 4 days (**a**) and 8 days (**b**) in growth medium. The cells were immunostained for Pax7, MyoD1, MyoG, and Desmin. Percentages of positive cells in each sample are presented as Box-Whisker plots with the median and the maximum 1.5 of the interquartile range (Q1-Q3). Outliers are included in the figure as circles but were excluded from statistical analysis. All populations expressed the selected myogenic markers, whereas a higher proportion of cells were positive for MyoG and Desmin, which are markers for terminally committed myogenic cells. Statistical analysis was performed by ANOVA (with the Holm-Sidak method as a pairwise multiple comparison procedure) or the Kruskal-Wallis One-Way ANOVA on Ranks (with Dunn’s method as a pairwise multiple comparison procedure), *p ≤ 0.05, **p ≤ 0.01, ***p ≤ 0.001, *n* = 4–9 animals

All data are presented as means ± standard deviation (SD), except in Figs. 2 and 4b which show Box-Whisker plots with median and 25%/75% quartiles. Statistical analyses were performed by using SigmaPlot 13.0 (Systat Software Inc.). All data sets were tested for normality (Shapiro-Wilk) and equal variance (Brown-Forsythe). If the tests for normality and equal variances were passed, the statistical significance of the data was assessed by Student’s t-test (Fig. 1b) or, for the comparison of all three subpopulations, by One-Way ANOVA followed by the Holm-Sidak method as a pairwise comparison test. If the normality test or the equal variance test failed, Kruskal-Wallis One-Way ANOVA on Ranks was performed with the Tukey test (equal sampling) or Dunn’s method (unequal sampling) as a pairwise comparison procedure. A *p*-value of ≤0.05 was considered to be statistically significant.

## Results

### Separation of SC/MPC subpopulations by various Percoll density gradients

SC isolation and enrichment by tissue digestion with 0.25% trypsin followed by Percoll gradient centrifugation were performed according to Mau et al. [23] with the exception that the Percoll gradient was changed from 25%, 40%, 90% to 25%, 40%, 70% (gradient 1) to remove erythrocytes. The protocol of Mau et al. has been shown to achieve SC-derived myoblasts cultures of high purity [23]. Nevertheless, primary isolates of muscle-derived cells are highly heterogeneous and can contain SC and MPC from different lineages and at variable developmental stages [31]. To consider the heterogeneity of SC/MPC, the mixed myogenic population (mixed P40/70) isolated by gradient 1 was split up into two subpopulations (SP40/50 and SP50/70) by introducing an additional 50% Percoll layer between the 40% and 70% Percoll layers (gradient 2, see Fig. 1a).

Indeed, directly after isolation, the total cell yield of SP40/50 and SP50/70 together was similar to that of mixed P40/70 (Fig. 1b). However, freshly isolated SP40/50 and SP50/70 accounted for 34% and 66% of the total cell yield, respectively. Furthermore, SP40/50 showed a significantly higher cell viability and cell size compared with mixed P40/70 and SP50/70.

To characterize the identity/purity and to assess the cell fate of the isolated cells, the expression of several myogenic markers was analyzed by flow cytometry during culture (Fig. 2a and b). SC and committed MPC are characterized by the expression of the transcription factors Pax7 and MyoD1, whereas terminally committed MPC are characterized by MyoG and Desmin expression [50]. Over the three populations, Pax7 and MyoD1 were expressed in approximately 12–30% and 6–13% of the cells, whereas MyoG and Desmin were expressed in approximately 45–63% and 18–32% of the cells at day 4 of culture (Fig. 2a). At day 8 of culture, the percentage of Pax7+ and MyoD1+ cells decreased in all populations compared with day 4, whereas the percentage of MyoG+ and Desmin+ cells increased, resulting in 87–94% MyoG+ and 60–66% Desmin+ cells (Fig. 2b). Looking at differences between mixed P40/70, SP40/50, and SP50/70 at day 4 of culture, we found that the proportion of Pax7+ cells was significantly higher in SP50/70 compared with mixed P40/70 and SP40/50 (Fig. 2a). In addition, a difference in the proportion of MyoD1 was evident between SP40/50 and SP50/70. During culture, the proportion of Pax7+ cells decreased in mixed P40/70 and in SP50/70, and simultaneously, more SP40/50 cells were MyoG+ in comparison with mixed P40/70 and with SP50/70 (Fig. 2b).

### Adhesion and proliferation properties of isolated SC/MPC populations

**Fig. 3:**
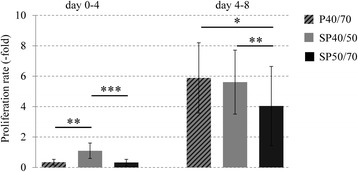
Proliferation rates of isolated SC/MPC populations. Proliferation rates of isolated populations from SM and LD muscles were calculated by using the changes in cell number between passaging (from day 0–4 and day 4–8 after isolation). SP40/50 showed significantly higher proliferation rates between day 0–4, in comparison with mixed P40/70 and SP50/70. The differences in proliferation between SP40/50 and mixed P40/70 disappeared between day 4–8, whereas SP50/70 retained lower proliferation rates than SP40/50 and mixed P40/70. Data are presented as means ± SD, *n* ≥ 10 animals. Statistical analysis of differences in proliferation was performed with a Kruskal-Wallis One-Way ANOVA on Ranks followed by a pairwise multiple comparison procedure (Dunn’s method), and significant differences are shown (*p ≤ 0.05, **p ≤ 0.01, ***p ≤ 0.001)

To quantify possible functional differences between mixed P40/70 and the subpopulations, proliferation rates were determined between day 0 and day 4 and between day 4 and day 8 of culture. In the period from day 0 to day 4, the proliferation rate of SP40/50 was significantly higher (3.2-fold), whereas the proliferation rate of SP50/70 (0.9-fold) was similar to that of mixed P40/70 (Fig. 3). Between day 4–8, the differences between mixed P40/70 and SP40/50 disappeared, but the proliferation rate of SP50/70 was still significantly reduced compared with mixed P40/70 and SP40/50.

**Fig. 4:**
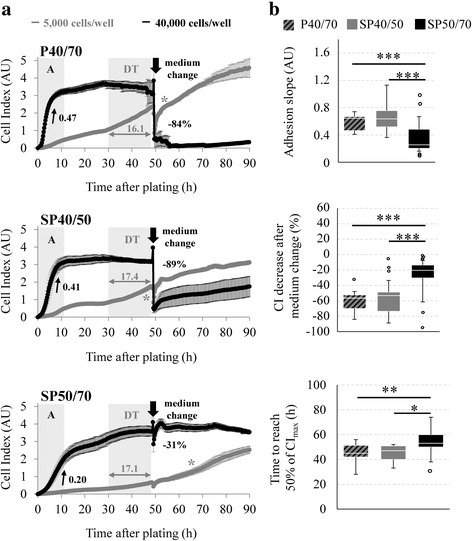
Growth curves of isolated SC/MPC populations. **a** Representative original growth curves of cells from mixed P40/70, SP40/50, and SP50/70 started at day 8 of culture. The impedance-based xCELLigence system was used for real-time monitoring of adhesion and proliferation of SC populations seeded at densities of 5000 cells per well (gray) or 40,000 cells per well (black), respectively. In adhesion assays, growth curves were analyzed regarding the slope during the adhesion phase (A) and the CI decrease after medium change. In proliferation assays, growth curves were analyzed with regard to the doubling time (DT) during the logarithmic growth phase and the time for CI to reach half of its maximum value (50% CI, marked with *). Curves were obtained from quadruplicates measured in parallel for each condition, and representative results are shown as means ± SD. **b** Box-Whisker plots are shown for selected growth parameters and include all experiments performed (*n* = 5–12 animals). Whiskers are presented with the maximum 1.5 of the interquartile range (Q_1_ – Q_3_), and the resulting outliers are included as circles. Statistical analysis of differences in growth parameters was performed by an ANOVA (adhesion slope, 50% of CI_max_) or Kruskal-Wallis One-Way ANOVA on Ranks (CI decrease after medium change) followed by a pairwise comparison test, and significant differences are marked with asterisks (*p ≤ 0.05, **p ≤ 0.01, ***p < 0.001). All populations showed characteristic kinetic profiles, but measurable differences occurred between them. The slope during cell adhesion is significantly steeper in mixed P40/70 and SP40/50 in comparison with SP50/70. The CI of mixed P40/70 and SP40/50 is influenced negatively by medium change, but significantly less dramatically in SP50/70. The time needed to reach 50% of the maximum CI (50% of CI_max_) value was different among the populations, whereas SP50/70 showed significantly higher values than mixed P40/70 and SP40/50

To investigate cell behavior in more detail, real-time growth curves for mixed P40/70 and the subpopulations were continuously recorded over a period of 90 h by the xCELLigence system. This system measures impedance changes caused by the presence of cells and calculates them as the dimensionless Cell Index (CI). Impedance readings are affected by the number of cells, the cell size, and the quality of their attachment and spreading, but changes in CI correlate with cell number throughout the experiment as has been confirmed by classic endpoint measurements [51, 52]. At the beginning of the experiment, cell size did not differ between the populations (mixed P40/70: 15.0 ± 0.73 μm, SP40/50: 14.9 ± 0.63 μm, SP50/70: 14.8 ± 0.71 μm). Representative original growth curves are given in Fig. 4a, and growth parameters from all experiments performed are summarized in Fig. 4b. In previous experiments, 5000 cells per well were selected as being the most suitable cell number to assess proliferation (proliferation assay). As adhesion is an important requirement for proliferation of cells, adhesion and spreading were also analyzed. Since a higher signal resolution is required for this experiment, adhesion assays were carried out with 40,000 cells per well.

In adhesion assays (black curves in Fig. 4a), the CI increased dramatically after seeding and reached a plateau after ~ 8 h in all populations. However, the adhesion slope was significantly higher in mixed P40/70 (0.59 ± 0.13) and SP40/50 (0.66 ± 0.21) in comparison with SP50/70 (0.38 ± 0.25) (Fig. 4b). Interestingly, we observed that mixed P40/70 and SP40/50 were more sensitive to a mechanical stress induced by medium change in comparison with SP50/70 (Fig. 4a and b). This was reflected by a sharp decrease of the CI (− 57% for mixed P40/70, − 49% for SP40/50, and − 26% for SP50/70), indicating that a higher proportion of cells from mixed P40/70 and SP40/50 were rather weakly attached to the bottom of the well.

In proliferation assays (gray curves in Fig. 4a), the proliferative capacity was determined by calculating the doubling time (DT) and the time to reach half of the maximal CI value (50% of CI_max_). The DT was similar for all three populations (mixed P40/70: 21.9 ± 6.4 h, SP40/50: 19.6 ± 3.6 h, SP50/70: 20.0 ± 4.0 h), but cells of SP50/70 needed significantly longer to reach 50% of CI_max_ (Fig. 4b, 53 ± 11 h) compared with cells of mixed P40/70 (45 ± 9 h) and SP40/50 (45 ± 7 h). A CI decrease after medium change, especially in mixed P40/70 and in SP40/50, was also seen in proliferation assays (gray curves).

### Myogenic differentiation potential of isolated SC/MPC populations

**Fig. 5:**
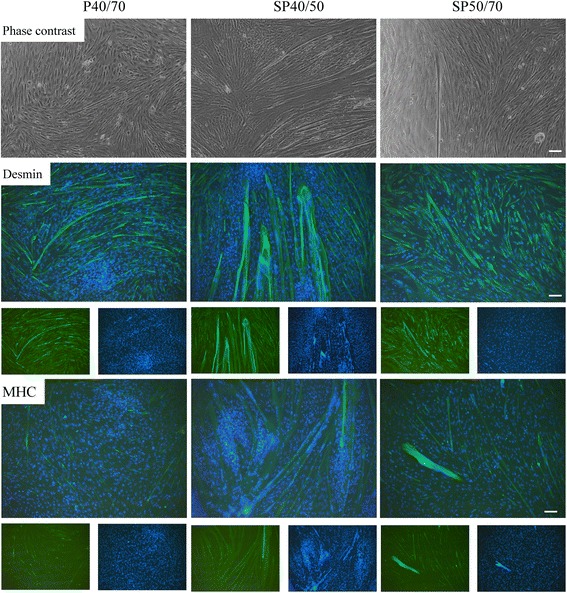
Differentiation potential of isolated SC/MPC populations. Isolated populations were seeded directly after isolation in growth medium, and before reaching confluence, cells were transferred to differentiation medium to allow myogenic differentiation. Differentiated cells were analyzed light microscopically and immunostained against Desmin and MHC to indicate their differentiation potential; cell nuclei were stained with DAPI. All populations were able to form multinucleated myotubes, whereas visually SP40/50 had the highest differentiation potential. The results are representative of three individual experiments from 3 animals. Scale bar represents 50 μm. See Fig. 6 for quantification

**Fig. 6:**
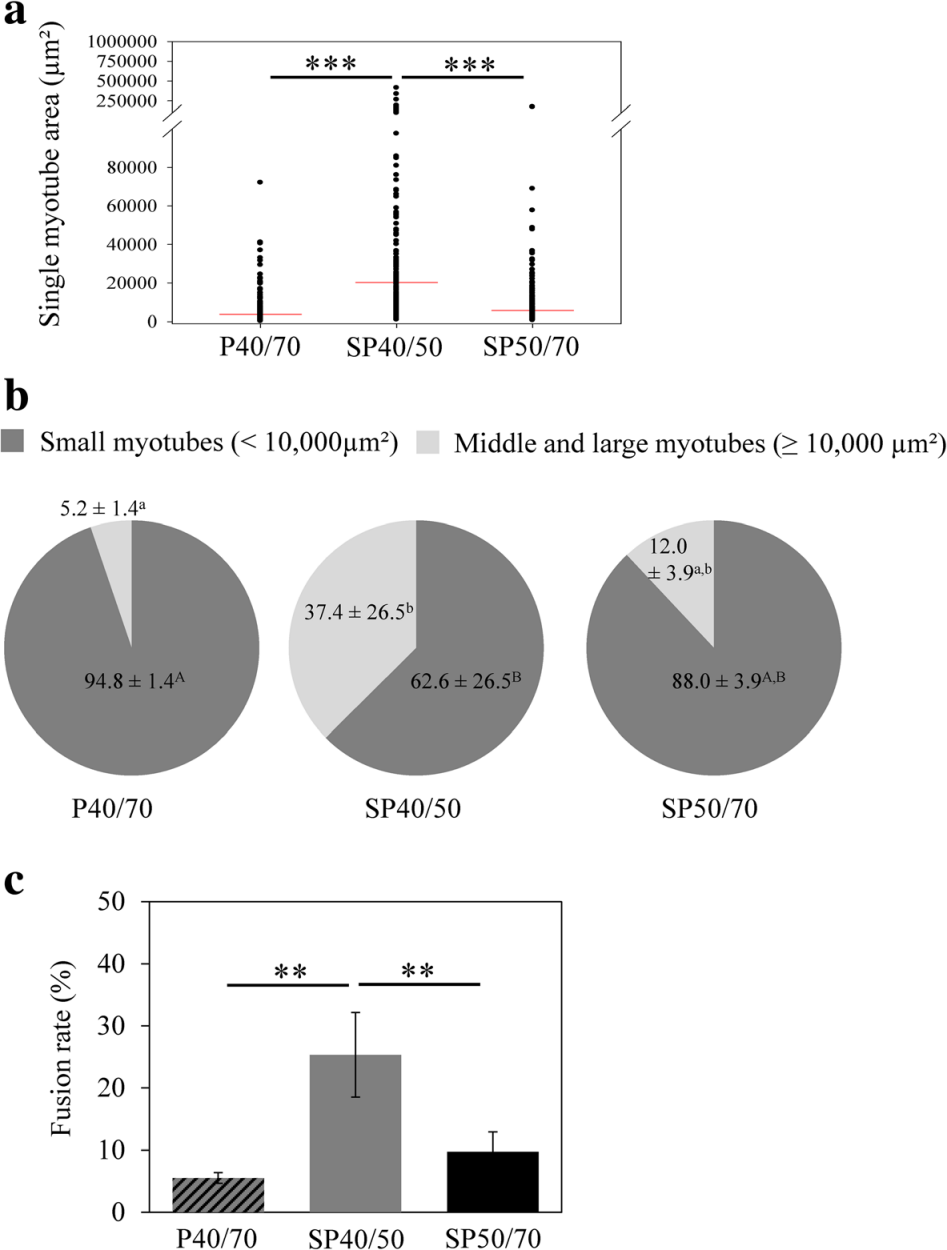
Myotube size distribution and fusion rate of differentiated SC/MPC populations. **a**, **b** Quantitative analysis of MHC immunofluorescence images to determine myotube area. **a** Each point represents the size of a single MHC+ myotube that contains two or more nuclei, and the horizontal line illustrates the mean value. **b** Myotubes with a size of < 10,000 μm^2^ were classified as small myotubes, whereas myotubes ≥10,000 μm^2^ were grouped as middle-sized and large myotubes. **c** Quantitative analysis of Desmin immunofluorescence images to determine fusion rate. SP40/50 contained more middle-sized and large myotubes, resulting in a significantly higher average myotube area in comparison with mixed P40/70 and SP50/70. Furthermore, significantly higher fusion rates were determined in SP40/50 compared with mixed P40/70 and SP50/70. The results represent three individual experiments from 3 animals. Statistical analysis was performed by a One-Way ANOVA (fusion rate) or Kruskal-Wallis One-Way ANOVA on Ranks (myotube area and distribution) followed by the corresponding pairwise comparison test, and significant differences are marked with asterisks in Fig. 6a and c (**p ≤ 0.01, ***p ≤ 0.001) or letters in fig. 6b (p ≤ 0.05)

As differentiation is a prerequisite for forming functional myogenic tissue, the differentiation potential of mixed P40/70 and the subpopulations was tested in vitro. Freshly isolated cells were cultured in growth medium, and before reaching confluence, medium was changed to serum-reduced differentiation medium. Mixed P40/70 and both subpopulations, SP40/50 and SP50/70, showed the ability to differentiate into the myogenic lineage, as they fused to elongated multinucleated myotubes, which were clearly visible in phase contrast images and images from Desmin- or MHC-stained cultures (Fig. 5). However, significant quantitative differences were found between the populations (Figs. 5 and 6) with SP40/50 exhibiting the highest differentiation capacity followed by SP50/70 and mixed P40/70. The myotube-covered area, determined by the MHC signal positive area, was markedly higher in SP40/50 (18,332 ± 17,897 μm^2^) than in SP50/70 (5816 ± 1148 μm^2^) and mixed P40/70 (4121 ± 1432 μm^2^, Fig. 6a) cultures. In addition, compared with mixed P40/70 and SP50/70 cultures, a higher proportion of middle-sized and large myotubes (≥ 10,000 μm^2^) formed in SP40/50 cultures (Fig. 6b), in accordance with the significantly higher fusion rate in SP40/50 cultures (Fig. 6c).

## Discussion

In this study, we present an easily feasible method for separating two functionally divergent SC/MPC subpopulations from a mixed population of myogenic cells isolated from postnatal piglet muscles. As described by others [23, 28, 46], SC were released from muscle tissue by enzymatic digestion with 0.25% trypsin, and thereafter, a Percoll density gradient (25%, 40%, 70%) was used to enrich SC/MPC at the 40/70% interface and to remove debris and non-myogenic cells such as fibrocytes [53] and in particular erythrocytes [46] from cell isolates. To obtain more information about the heterogeneity and interaction of SC/MPC, we divided the mixed 40/70 population into the 40/50 and 50/70 subpopulations by using a more stepped 25%, 40%, 50%, 70% Percoll gradient. The resulting subpopulations were characterized regarding their myogenic marker profiles and their functional behavior (adhesion, proliferation, and differentiation).

The proportion of myogenic cells was approved by the expression of Pax7 and/or MyoD1, and particularly by the high proportions of early differentiated, MyoG+ cells in day 4 (up to 63%) and day 8 (≥ 90%) cultures held under growth-promoting conditions. The marker expression analysis revealed that all isolated cell populations had a high degree of myogenic purity. Moreover, differentiation assays confirmed their ability to differentiate into the myogenic lineage. In several studies, Desmin rather than MyoG has been used to analyze the purity of SC isolates from piglet muscles [23, 54, 55]. Although the proportion of Desmin+ cells significantly increased from an average of 25% to 63% during culture in our study, it was clearly below the values observed for MyoG. The presence of Desmin during myogenic commitment has been shown to vary between rodents, chicken, and cattle [56, 57], and the temporal and spatial expression pattern of Desmin has not been precisely described in pig. Hence, we cannot exclude that Desmin is expressed later than MyoG during myogenesis in SC/MPC isolated from neonatal piglets; this would explain the lower proportion of Desmin+ cells than MyoG+ cells in our cell populations. It should be noted that the absence of Desmin does not automatically point to a non-myogenic cell. Therefore, we consider that MyoG is a more useful myogenic marker for our cell isolates.

Analysis of myogenic marker expression revealed that our myogenic cell isolates contained a mixture of Pax7+ immature cells, MyoD+ myogenic progenitor cells/myoblasts and further committed MyoG+ myocytes. During culture, they were able to form myotubes. Our results are in accordance with results showing that the population of mononuclear cells in the muscle is heterogeneous during early postnatal growth [58]. These cells are pre-quiescent and pass through distinct cell states during myogenic commitment accompanied by a characteristic time-dependent myogenic marker expression profile [36, 59, 60]. Whereas only 2–5% of myonuclei belong to SC in the adult stage, a much higher proportion (60% in the pig) has been found during the early postnatal period, of which a large number (90%) is in the cycling state [5, 9]. This proliferating population mainly consists of Pax7+/MyoD1+ cells and, to a lesser extent, of Pax7+/Myf5+ and MyoG+ cells marked for terminal differentiation [5, 18, 58, 61]. This proliferative phase is necessary to generate progeny that differentiate and supply nuclei for growing myofibers. Simultaneously, increasing numbers of quiescent cells with self-renewal potential emerge [58].

During postnatal growth, a SC pool is formed that shows marked molecular and functional heterogeneity, including the different expression of myogenic markers and the occurrence of distinct high- and low-dividing subpopulations [34, 37, 40]. In our study, the 4 day-old SP50/70 cultures differ from the SP40/50 and mixed P40/70 cultures by having a higher proportion of Pax7+ and MyoD1+ cells. As Pax7 has been shown to play a critical role in the early postnatal survival and expansion of SC [16, 62], this result is in accord with the dominance of SP50/70 (66%) over SP40/50 (34%) in freshly isolated cells. Moreover, Pax7 directs myogenic progenitors to withdraw from myogenic differentiation and to pass into a more dormant state, a prerequisite for their transition into the adult quiescent SC type [15, 63]. From our results, we can hypothesize that, during early postnatal muscle development, the dominant SP50/70 contributes markedly to the formation of the reserve cell/SC pool. Compared with early day 4 cultures, the proportion of Pax7+ cells decreases in day 8 cultures of mixed P40/70 and SP50/70, whereas it remains at about 10% in SP40/50.

Furthermore, the real-time monitoring of the adhesive behavior and complete growth kinetics of SC populations has revealed that SP40/50 and mixed P40/70 constitute a fast adhering and fast proliferating phenotype, whereas SP50/70 cells show considerably slower adhesion and exhibit a slow proliferation phenotype. A link between the adhesion ability and proliferation capacity of muscle-derived cells from turkey and human has also been found by Rouger et al. and Sellathurai et al. [12, 45]. Low- or non-adherent conditions are known to decrease the activation capacity of cells, to slow or arrest the cell cycle, and to reduce the activity of the main growth-factor-depending signaling pathways [27, 64–67]. In further experiments, an examination of whether cell attachment signaling pathways (e.g., integrin, focal adhesion kinase) and other adhesion molecules (e.g., laminin, N-CAM, and M-cadherin) contribute to the different adhesion/proliferation behavior of the separated subpopulations will be of interest.

Some of our results suggest the possible interaction between the cells of the two subpopulations. SC are embedded in their environmental niche and interact with their neighboring cells. As has recently been shown, SC interact with macrophages and endothelial cells, resulting in the modulation of SC functionality, e.g., proliferation and differentiation [68–70]. SP40/50 has a higher proliferation and differentiation potential than mixed P40/70 and SP50/70. In accord, the percentage of MyoG+ cells in SP40/50 increases above that of SP50/70 and mixed P40/70 during culture, and SP 40/50 cells show a higher fusion rate and form a higher proportion of middle-sized and large myotubes. In other studies by Rouger et al. and Schultz [9, 37], fast-dividing SC populations also had a higher differentiation potential. In our study, the proliferation rates of mixed P40/70 and SP40/50 equalize during culture, whereas the differentiation behavior of mixed P40/70 exhibits more similarity to that of the slow-proliferating SP50/70 cells. Furthermore, compared with SP50/70, mixed P40/70 and, to a lesser extent, SP40/50 show a higher sensitivity to mechanical stress induced by medium change reflecting weaker adhesion to the surface. A sharp CI drop after medium change has also been observed in xCELLigence growth kinetic assays performed with other cells types, such as gingival fibroblasts and breast cancer stem cells [71, 72]. Since mixed P40/70 includes cells of SP40/50 and SP50/70 (see Fig. 1a), we suggest that the latter trigger the adhesion behavior of SP40/50. However, until now, very little work has been carried out regarding cell communication and/or interaction between the various myogenic precursor cells. Thus, an analysis of the impact and the direction of possible interactions between SC populations will be of interest in future studies.

## Conclusions

This study demonstrates that a discontinuous Percoll density gradient centrifugation is a useful tool for subdividing myogenic cell populations from perinatal porcine muscle into functionally divergent subpopulations. This approach allows SC heterogeneity to be analyzed to a greater extent, giving a better understanding of the way that individual cell populations are involved in the postnatal development and plasticity of skeletal muscle of pigs. Since the separation of subpopulations is based solely on differences in cell densities, we consider that this method, possibly with minor modifications, will also be applicable for other species.

## Abbreviations

CI: Cell Index
DT: Doubling time
LD: *Musculus longissimus dorsi*
MPC: Myogenic precursor cells
SC: Satellite cells
SD: Standard deviation
SM: *Musculus semimembranosus*
